# Molecular Recognition of Proteins through Quantitative Force Maps at Single Molecule Level

**DOI:** 10.3390/biom12040594

**Published:** 2022-04-18

**Authors:** Carlos Marcuello, Rocío de Miguel, Anabel Lostao

**Affiliations:** 1 Instituto de Nanociencia y Materiales de Aragón (INMA), CSIC-Universidad de Zaragoza, 50018 Zaragoza, Spain; rociodmv@gmail.com; 2 Laboratorio de Microscopias Avanzadas (LMA), Universidad de Zaragoza, 50018 Zaragoza, Spain; 3 Fundación ARAID, 50018 Zaragoza, Spain

**Keywords:** adhesion maps, atomic force microscopy, jumping mode, molecular recognition imaging, rupture force, protein:ligand interactions, single molecule, protein detection

## Abstract

Intermittent jumping force is an operational atomic-force microscopy mode that produces simultaneous topography and tip-sample maximum-adhesion images based on force spectroscopy. In this work, the operation conditions have been implemented scanning in a repulsive regime and applying very low forces, thus avoiding unspecific tip-sample forces. Remarkably, adhesion images give only specific rupture events, becoming qualitative and quantitative molecular recognition maps obtained at reasonably fast rates, which is a great advantage compared to the force–volume modes. This procedure has been used to go further in discriminating between two similar protein molecules, avidin and streptavidin, in hybrid samples. The adhesion maps generated scanning with biotinylated probes showed features identified as avidin molecules, in the range of 40–80 pN; meanwhile, streptavidin molecules rendered 120–170 pN at the selected working conditions. The gathered results evidence that repulsive jumping force mode applying very small forces allows the identification of biomolecules through the specific rupture forces of the complexes and could serve to identify receptors on membranes or samples or be applied to design ultrasensitive detection technologies.

## 1. Introduction

Molecular recognition is today one of the most important analytical challenges. Traditionally, it consists in the determination of elemental or molecular composition by sensitive analysis tools, but the increasing need to identify smaller matter quantities requires the development of new methods. Scanning probe microscopies (SPM) with imaging capabilities down to the molecular or atomic scale [1] have raised hopes that it may be possible to determine atomic or molecular composition with a comparable resolution. Chemical analysis can be achieved using a scanning tunnelling microscope through spectroscopy of electronic states [2]. However, for non-conducting biological material that must be scanned mimicking physiological conditions, remarkable data on electronic states are not expected. On the contrary, among SPMs, atomic force microscopy (AFM) has become a powerful tool for imaging and measuring physical parameters at the single-molecule level [3,4], allowing atomic chemical identification under certain conditions [5].

Nowadays, AFM has emerged as a cutting-edge tool to provide data on the topology, adhesion, elasticity, dynamics, conductivity and other properties of biomolecular samples, and is currently the most-used approach for force spectroscopy (FS) studies [6,7]. AFM imaging may characterize molecules based on morphological, electrostatic, magnetic, optical or mechanical properties. Particularly interesting for biosystems is nanomechanical characterization. Intra- and intermolecular interaction forces can be accurately measured by AFM-FS at near-physiological conditions with piconewton sensitivity [8]. In AFM-FS experiments, the functionalized AFM tip with the ligand of interest is moved directly towards a nanoflat rigid surface where a complementary receptor is located, or vice versa. Once the AFM tip makes contact with the external sample surface, it is retracted, measuring the intermolecular interaction between the functionalized AFM tip and the biomolecular sample by a so-called force–distance or Fz curve. This action may then be repeated to provide full statistical information regarding the intermolecular unbounding interactions. Appropriate non-destructive immobilization methods are therefore required [9]. Going beyond, the use of dynamic force spectroscopy (DFS) based on the Bell-Evans (BE) theoretical framework has become a powerful analytical method to explore the energy landscape of the ligand-receptor unbinding processes [10,11,12]. DFS provides mechanostability information about the complex formed by measuring its rupture force at different loading rates [11]. FS has been used to study numerous biosystems, including antigen/antibody [13,14], glycoproteins/carbohydrates [15], integrin/fibronectin [16], DNA/peptides [17], lignocellulosic polymers [18], flavoenzymes [19] and enzyme/coenzyme [20], among others. These investigations have revealed insights into fundamental biochemical processes and enabled the development of analytical devices [21].

Today, recognition studies with AFM can be developed using different methods. The pioneer work patented in this field was called force–volume (F–V) mode, which consists of the simultaneous acquisition of topography images and force-distance curves [22]. Numerous biological questions have been addressed by this F–V operational mode, and the store of full Fz at each image pixel renders very long acquisition times. An alternative approach was developed by Hinterdorfer et al. named simultaneous topography and recognition imaging (TREC) [23,24]. TREC consists of a feedback loop which allows to discriminate topographical contributions from recognition events in the AFM cantilever motion. Another alternative is tuning-fork-based transverse dynamic force microscopy (TDFM) [25], which relates topography images of, e.g., avidin with the phase signal of the tuning fork oscillation used to detect single molecular-recognition events, while changes in the lateral amplitude were used as the feedback parameter for topographical imaging using a biotinylated tip as a sensor. The main drawback of the aforementioned TREC and TDFM approaches is the lack of quantitative information provided by the phase maps, which limits the resolution to discriminate the biomolecule entities present on mixture systems. To overcome all the above-described limitations, jumping mode (JM) was used as a starting point. JM enables the acquisition of a sequence of force–distance curves at each defined point of the sample surface [26]. JM only records the maximum adhesion force from the Fz curves instead of the entire full force profile as F–V mode does. This aspect significantly minimizes the data-acquisition time.

In this work, JM was implemented operating in a repulsive regime applying very low forces with functionalized tips, which protects from unspecific tip-sample forces; thus, the adhesion maps become molecular-recognition maps. The aim of the work is not only to recognize or locate receptors on a sample surface but goes further to unequivocally identify and distinguish between similar proteins on a mixture sample through scanning with complementary ligand functionalized tips on adhesion maps. Here, we demonstrate the high-potential applications of the present method being capable to differentiate among avidin and streptavidin on a hybrid sample, which bind the same ligand through the strongest non-covalent interaction known in nature. Biotin ligand was attached to the AFM tips, while the two proteins are indistinguishable in the topography images due to their protein structures being very similar. The concept is to characterize the different rupture-force maps of the two proteins separately for further identification on hybrid samples in fast and controlled conditions. This method may be applied to locate proteins or other biomolecules on substrates or microorganism membranes, avoiding false events. Additionally, as the strept(avidin)–biotin system has become one of the most exploited systems for biochemical analysis, this method could be used to locate receptors previously labeled with them. Proteins immobilized onto solid surfaces exhibit potential applications in matters as varied as biosensors [27,28,29] or drug screening [30,31], so this method could be used as a basis to develop high-throughput devices for drug screening or lab-on-a-chip, among others.

## 2. Materials and Methods

### 2.1. Protein Labeling and Covalent Immobilization on Mica

**Figure 1:**
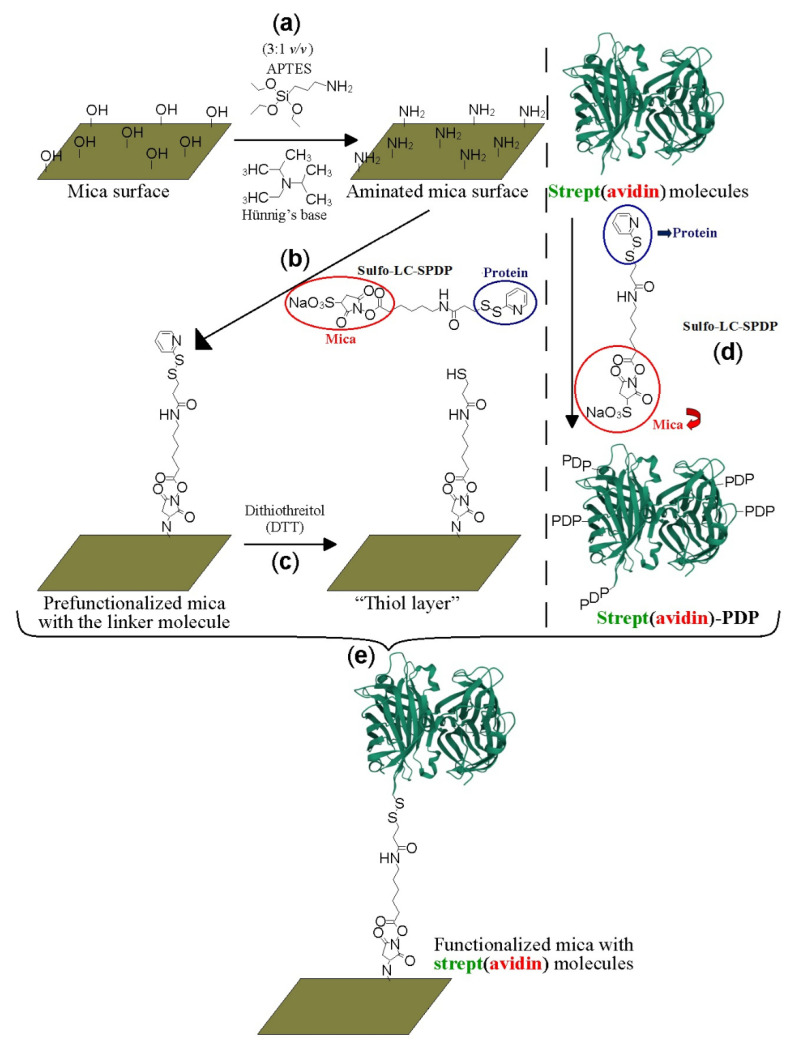
Schematic representation of the covalent mica functionalization with strept(avidin) protein molecules. Amination of the mica surfaces (**a**), reaction of the aminated mica surface with the Sulfo-LC-SPDP linker molecules (**b**), reduction with dithiothreitol agent to expose reactive sulfhydryl groups (**c**), labeling of the strept(avidin) protein molecules with the Sulfo-LC-SPDP crosslinker molecules, (**d**) and final reaction between the prefunctionalized mica surfaces with the tagged strept(avidin) protein molecules via disulfide bonds (**e**).

Freshly cleaved mica pieces (Electron Microscopy Sciences; Hatfield, UK) were exposed to 3-aminopropyl triethoxysilane (APTES; Sigma-Aldrich, San Luis, MO, USA) and N,N-diisopropylethylamine (Hünig’s base; Sigma-Aldrich, San Luis, MO, USA) (1:3 *v/v*) in gas phase under an argon atmosphere for 2 h (Figure 1a). Subsequently, each aminated mica piece reacted with 20 mM sulfosuccinimidyl 6-(3′-[2-pyridyldithio]propionamido) hexanoate (Sulfo-LC-SPDP; Thermo Scientific Pierce, Waltham, MA, USA) heterobifunctional linker molecule in PBS/EDTA-azide for 50 min at room temperature (Figure 1b). Mica-PDP was reduced with freshly prepared 150 mM dithiothreitol (DTT; Sigma-Aldrich, San Luis, MO, USA) for 30 min at 4 °C in 50 mM PBS/EDTA-azide under stirring to expose sulfhydryl groups towards the solution (Figure 1c). Sideways, avidin and streptavidin proteins (Sigma-Aldrich, San Luis, MO, USA) were incubated with 20 mM Sulfo-LC-SPDP for 50 min at 4 °C (Figure 1d). Lysine amine groups of the proteins reacted through the N-hydroxisuccinimide (NHS) moiety of SPDP creating amide bonds [32].

The randomly decorated proteins-PDP (where PDP represents the tag from the pyridyldithiopropionamide part of the cross-linker used) were purified using PD-10 desalting columns (GE Healthcare, Chicago, IL, USA) in 50 mM PBS-EDTA-azide. The functionalized proteins were attached on 1 cm^2^ of chemically modified muscovite mica sheets treated as described above (Figure 1e). Immobilized samples were prepared with separate protein molecules, i.e., without overloading all reactive sulfhydryl groups. Mica pieces with different concentrations (0, 0.2, 0.5, 1.0, and 3.0 µg/mL) of avidin or streptavidin and also hybrid with both proteins were prepared. Avidin-PDP and streptavidin-PDP species were incubated for 18 h under stirring on the thiol-terminated mica pieces and reacted covalently to form disulfide bonds between them [32]. The samples were extensively washed with 50 mM PBS, 0.2% Tween 20 (Panreac Química SLU, Castellar del Vallés, Spain), 0.1% SDS (Panreac Química SLU, Castellar del Vallés, Spain) for 30 min with gentle stirring, and finally, with 50 mM PBS pH 7.4 to release the loosely bound proteins on the mica surface that could negatively interfere during image acquisition. Samples with isolated protein molecules are required to better appreciate their molecular identification at the single-molecule level.

### 2.2. HRP–Biotin Enzymatic Assays

The functionality of the immobilized proteins was verified by a colorimetric-specific assay in the presence of biotinylated horseradish peroxidase (biotin–HRP; Thermo Scientific, Waltham, MA, USA). Prior the incubation of the proteins on the mica surface, two calibration experiments were conducted. Measurements of protein samples both in solution and immobilized on the mica surface were performed. This assay is based on the enzymatic reaction between biotin–HRP and 3, 3′, 5, 5′ tetramethylbenzidine chromogen (TMB) when it is oxidized in the presence of hydrogen peroxide (H_2_O_2_) [33]. This redox reaction causes a color change in the medium from colorless to blue and from blue to yellow when the reactivity is stopped by the addition of sulfuric acid (Panreac Química SLU, Castellar del Vallés, Spain).

Thus, by following the absorbance at 450 nm of the final solution, it is possible to precisely monitor the amount of bound biotin, and thus, the functional amount of strept(avidin) at the surface. TMB and H_2_O_2_ were part of the commercial kit TMB Substrate Kit (Thermo Scientific, Waltham, MA, USA). Calibration curves for two series of solutions were developed using 0, 0.19, 0.39, 0.78, 1.56, and 3.12 ng/mL biotin–HRP. 0.5 µg/mL strept(avidin) and 400 µL of 0.52 mM TMB solution (composed of H_2_O_2_ and peroxidase substrate at equal parts) diluted in PBS-EDTA buffer were added and incubated for 5 min. Then, 100 µL of sulfuric acid concentrate (Sigma-Aldrich, San Luis, MO, USA) was added to stop the enzymatic reaction. The absorbance of the resulting solutions was measured at 450 nm with a Cary 100 Bio spectrophotometer (Agilent Technologies, Santa Clara, CA, USA).

Each piece of mica containing the protein of interest was attached through vacuum grease to a 6-well ELISA Nunclon Surface (Thermo Scientific Nunclon, Waltham, MA, USA). A measure of 200 µL of 100 µM biotin–HRP (concentration in excess) was added to each substrate and incubated for 30 min. To remove unbound enzyme, the mica surfaces were washed successively with PBS, 0.2% Tween 20 (Panreac Química SLU, Castellar del Vallés, Spain), 0.1% SDS (Panreac Química SLU, Castellar del Vallés, Spain) under mild stirring for 30 min. Then, the mica surfaces were gently washed with PBS-EDTA to remove the detergent molecules. For colorimetric measurements of the immobilized proteins, 1.5 mL of 0.5 mM TMB was added to each well and incubated under stirring for 5 min. During this time, it should be noted that the color of the solution changed from colorless to blue. If the color changes from blue to green, the measurement is invalid since there is an excess of protein concentration outside the range of the calibration curve. The enzymatic reaction is stopped as described above by adding 100 µL of sulfuric acid concentrate in each well while being gently stirred. During this step, a color change of the media from blue to yellow should be observed. To read the absorbance at 450 nm, 1.2 mL of PBS was added to each well to reach the optimal cuvette volume to conduct spectrophotometry measurements. The resulting data were exported to the previously calculated calibration curves to quantify the amount of strept(avidin) molecules immobilized on the mica surface upon their incubation.

### 2.3. Topography and Adhesion AFM Mapping Analysis

AFM measurements were performed with the jumping mode (JM) in a Cervantes FullMode SPM system (Nanotec Electrónica, Madrid, Spain). JM works by performing a force scan at each image point of the sample surface, the feedback signal being the loading normal force. Sinusoidal voltage waves are applied by the scanning piezoelectric to minimize piezoelectric resonances. Maximum tip-sample adhesion is calculated by a digital signal processing (DSP) board for each force curve at any pixel of the image, and its value is registered together with the corresponding pixel height. Thus, this method allows the simultaneous acquisition of both topography and tip-sample maximum adhesion data relatively quickly [26]. Images were taken with bare V-shaped silicon nitride cantilevers with a spring constant of 0.03 N/m (MSNL Microlever; Bruker Probes, Santa Barbara, CA, USA). The nominal sharp tip radius was 2 nm, minimizing non-desirable broadening effects [34]. This effect is typically observed in AFM studies and mainly depends on the tip apex shape and sample morphology [35]. To calculate the percentage of species contained on the functionalized mica surface, several scan areas with different scan sizes were analyzed. Cross-section profiles of the features were recorded by magnifying the area without losing information [36]. This fact is due to the original resolution of the achieved AFM images being defined as 256 pixels/lines. A statistical study of the strept(avidin) heights was fulfilled, counting 25 protein features. The bin size was identically settled for both cases, being fixed at 0.1 nm in order to ensure the consistency of the statistical analysis. Adhesion imaging was performed using V-shaped silicon nitride cantilevers modified with a biotin-terminated polyethylene glycol (PEG) linker. The spring constant of the AFM levers and the length of the PEG spacer when fully stretched were 0.01–0.03 N/m and 20 nm, respectively (Novascan Technologies Inc., Ames, IA, USA).

Negative FS controls were conducted with unmodified AFM tips on strept(avidin) molecules to determine non-specific rupture forces. The intermediate functionalization step was also checked; thiolated mica was scratched, applying higher forces using rectangular cantilevers with a spring constant of 0.73 N/m (ORC8 microlever, Bruker Probes, Santa Barbara, CA, USA) to verify its quality; and it was also imaged with the biotin probes to observe possible adhesion events. Adhesion force scale bars were homogenized to 100 pN to better visualize differences in the respective interactions of strept(avidin)/thiol layers with biotinylated tips. Measurements were conducted in 50 mM PBS pH 7.4 at 20 °C, at a rate of 130 pixel/s. JM was operated under a repulsion regime [37] with the application of very low forces, in the order of 35 pN [38]. The applied force was optimized running several force–distance curves (N 10), selecting the force to work under the repulsive electrical double layer (REDL) [37]. REDL not only minimizes the non-desirable unspecific interactions between the functionalized AFM tip and the sample surface but also becomes negligible due to the frictional lateral forces exerted between the AFM tip edges and the biomolecules of interest. Raw image processing was performed with the WSxM software [39]. Adhesion images were calibrated with the stiffness data estimated from backward Fz curves taken at the same conditions. Rupture forces were estimated analyzing around 200 rupture events from different areas of several adhesion images. Force data were taken from the maximum adhesion of the force profiles plotted on the events of the adhesion maps corresponding to protein molecules of the topography maps. The adhesion force statistical analysis was conducted for the functionalized mica following all subsequent chemical steps, but in the absence of proteins (also called the thiol layer since mica exposes sulfhydryl groups at the external side). The bin size was defined as 1 pN for the thiol layer condition and 3 pN when the mica was decorated with strept(avidin) protein molecules. All adhesion force measurements were carried out at a defined loading rate of 1 nN/s.

## 3. Results

### 3.1. HRP–Biotin Enzymatic Assays

HRP–biotin enzymatic assays were conducted to determine the number of strept(avidin) molecules present per unit area of mica. For this purpose, color modifications of the chromogenic TMB substrate were monitored to discern the HRP enzymatic activity. Two different types of negative control were carried out to check whether the absorbance differences registered on the spectrophotometer are due to the HRP peroxidase reaction or based on side non-desirable reactions when the proteins are non-involved (Table 1).

**Figure 2:**
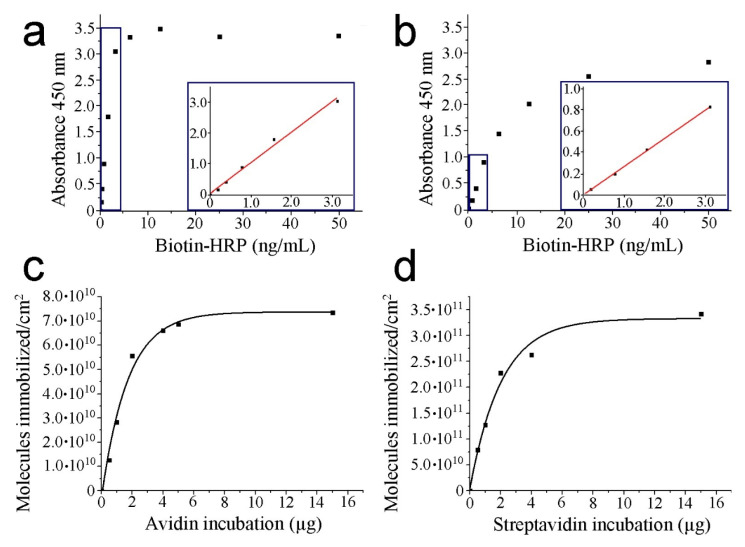
HRP–biotin enzymatic assays. The absorbance is due to the oxidation of TMB under the presence of H_2_O_2_ in PBS-EDTA buffer after incubation for 5 min. Sulfuric acid was added to stop the reaction. Calibration curves of avidin (**a**) and streptavidin (**b**), respectively. The inset figures (blue squares) depict a zoom of the curve region considered to perform the linear fitting. Representation of the attached molecules per area unit respect protein incubation for avidin (**c**) and streptavidin (**d**), respectively.

We demonstrate the significant signal detection of the pre-functionalized mica surfaces in the previous chemical step before strept(avidin) addition (absorbance mean value of 0.6367). This finding points out the need to settle blank measurements of thiol layers prior to strept(avidin) incubation to prevent overestimation of the immobilized protein amount. Moreover, the absence of the TMB chromogenic molecule was also tested. In this condition, negligible values of absorbance were recorded (absorbance mean values of 0.0030, 0.0024, and 0.0032 for thiol layers; 2 µg of avidin and streptavidin incubated, respectively) (Table 1). This observation demonstrates that the presence of TMB is required to act as a hydrogen donor for the reduction of H_2_O_2_ to water by HRP. Calibration curves were obtained by the incubation of biotin–HRP on the mica surface (from 0 to 50 ng/mL) (Figure 2a,b for avidin and streptavidin, respectively).

A plateau is observed indicating monolayer formation of biotin–HRP on mica surface (from 7.5 ng/mL and 40.0 ng/mL of biotin–HRP for avidin and streptavidin, respectively). For this reason, to extrapolate the absorbance, values below 3.0 ng/mL are used for both strept(avidin) proteins to remain at the linear regression (linear fitting provides equations of Abs_450_ = 1.184biotin–HRP(ng/mL)-0.028 with a regression coefficient of 0.99 and Abs_450_ = 0.265biotin–HRP(ng/mL) with a regression coefficient of 1.00 for avidin and streptavidin, respectively (insets of Figure 2a,b). The absorbance values at a wavelength of 450 nm from the calibration data may be directly related to the amount of biotin–HRP bound to the strept(avidin) proteins.

Despite presenting similar affinity constants (Ka ≈ 10^15^ M^−1^ and 10^14^ M^−1^ for avidin and streptavidin, respectively), the calibration lines calculated under the same conditions for the two proteins show slight differences. This fact may be due to structural and charge differences between strept(avidin) proteins, which will also be reflected in the results of the molecular recognition imaging experiments. Finally, different amounts of strept(avidin) are incubated (up to 15 µg) to calculate the number of molecules on the mica surface (Table 2).

To estimate the number of strept(avidin) molecules, it was hypothesized that each immobilized strept(avidin) protein binds two biotin ligands on average. The number of immobilized strept(avidin) molecules rises when the amount of incubated protein increases (Figure 2c,d for avidin and streptavidin, respectively). Streptavidin renders a higher number of attached molecules on the mica surface than avidin when the monolayer is formed (3.4 10^11^ vs. 7.4 10^10^ molecules, respectively). This fact could be due to the slightly minor dimensions of streptavidin monomers. Since the goal of this work is to perform molecular recognition imaging, lower incubation amounts of strept(avidin) may be used to prevent protein monolayer formation on the mica surface and, thus, to clear discriminate between the protein molecules. For this reason, the representations after exponential fitting show the convenience of incubating less than 5 µg for both strept(avidin) proteins to enable the visualization of single features for further AFM measurements (R-squares are 0.98 and 0.96 for avidin and streptavidin, respectively). Once the incubation of strept(avidin) amounts are fully optimized on mica, JM is exploited to collect force maps of samples based on one or two protein samples scanned with a biotin sensor.

### 3.2. AFM Measurements

**Figure 3:**
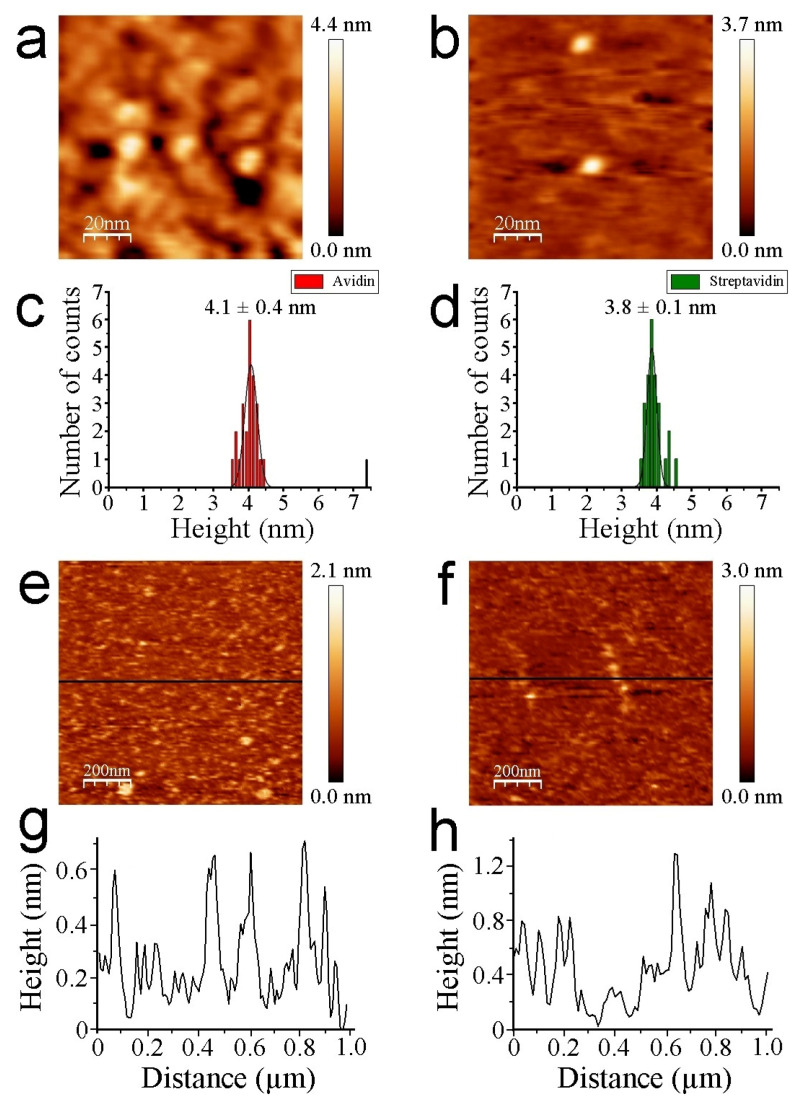
Representative AFM images of individual avidin (**a**) and streptavidin (**b**) protein molecules. Scan size is 100 nm × 100 nm. Histogram height analysis of avidin (**c**) and streptavidin molecules (**d**) (N 25). Topography of thiol monolayers (**e**) and a scratched area (**f**). Scan size is 1 µm × 1 µm. Cross-section profiles are depicted for aforementioned layers ((**g**) and (**h**), respectively). Black lines represent the region where the cross-section profiles are traced.

The thiol-layer substrate serves as a negative control, not only to check whether functionalization chemical steps on mica take place properly but also to discern potential interference effects during molecular image recognition. The analysis of single strept(avidin) proteins aids in better understanding and calibrating the interaction that is undergone with biotin AFM tips. Finally, the binary hybrid system of strept(avidin) to identify individual proteins at a nanoscale level will be analyzed. Operation in a non-contact REDL regime is crucial for two key aspects: (i) to keep the protein morphology intact, and thus, functionality; and (ii) to minimize the tip-sample mechanical contact, reducing the non-specific tip-sample interactions [37,40]. Strept(avidin) population features are mainly monomeric (single isolated tetramers), with the exception of a negligible amount of dimers. The Z heights of strept(avidin) estimated by AFM imaging are slightly lower than expected (Figure 3a,b for avidin and streptavidin, respectively).

**Figure 4:**
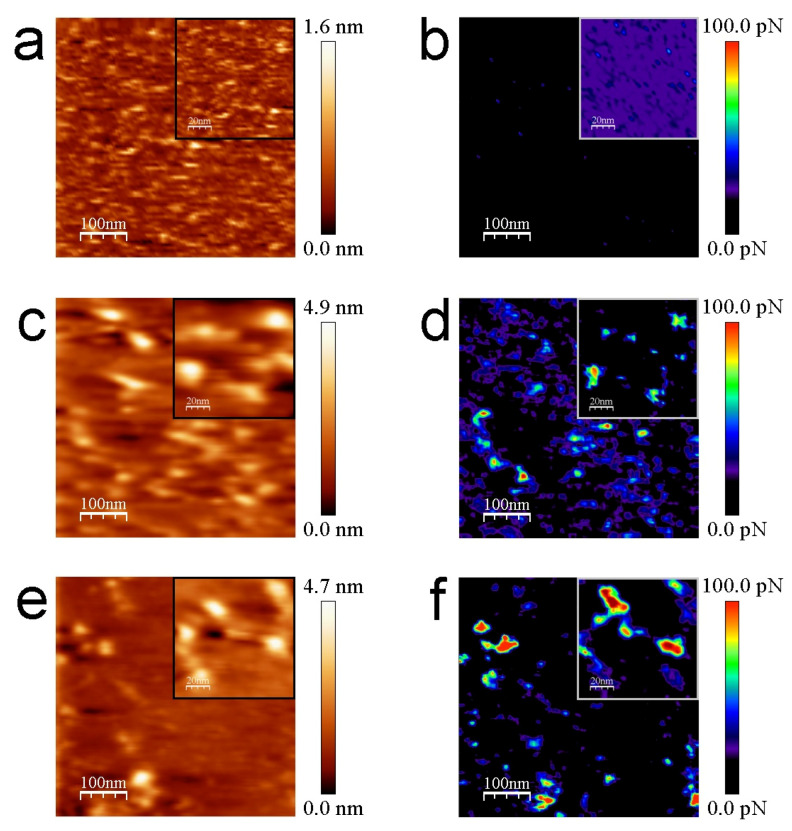
Representative molecular recognition images for thiols (**a**,**b**), avidin (**c**,**d**), and streptavidin films (**e**,**f**). Topography (**a**,**c**,**e**) and simultaneous adhesion (**b**,**d**,**f**) maps of the aforementioned layers provided with biotinylated tips. The inset figures represent the magnification of the studied thiol/strept(avidin) samples. Scan sizes are 500 nm × 500 nm for the bigger images and 100 nm × 100 nm for the insets.

The structure of avidin resolved by X-ray crystallography [41] shows similar dimensions with respect to streptavidin protein [42,43], rendering both molecules around 5 nm in diameter (PDB codes: 1VYO and 1SWA for avidin and streptavidin, respectively). This effect has been demonstrated to be undergone whilst working on the REDL regime [36]. Our strept(avidin) dimension data are consistent with previous studies [44,45]. The mean Z-height values are 4.1 ± 0.4 and 3.8 ± 0.1 nm for avidin and streptavidin protein molecules, respectively (Figure 3c,d, respectively). These data corroborate the similar morphology and dimensions of both studied proteins. Thiol layers render uniform surface chemistry (Figure 3e). Scratching measurements are carried out to ensure the quality and height of these functionalized mica surfaces (Figure 3f). Cross-section profiles show the homogeneity of the thiol layers in terms of Z-height (Figure 3g). Cross-section profiles from the scratched area of thiol layers depict with exactitude a height of approximately 0.5 nm (Figure 3h). These data are in agreement with previous works [32,46]. Molecular recognition imaging controls of thiols (Figure 4a,b), avidin (Figure 4c,d), and streptavidin (Figure 4e,f) samples were performed using biotinylated AFM tips.

**Figure 5:**
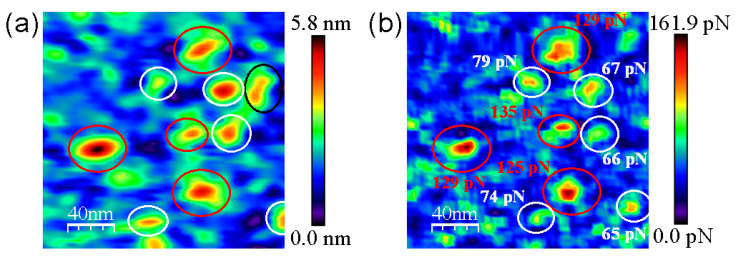
Representative molecular recognition images for the hybrid mixture of strept(avidin) proteins. Topography (**a**) and adhesion (**b**) maps given by scanning with biotinylated tips. Individual avidin and streptavidin protein molecules identified through the unbinding forces of the corresponding complexes with biotin displayed in (**b**) are shown in white and red, respectively. Non-identified protein features are shown in black circles. Scan size is 200 nm × 200 nm.

**Figure 6:**
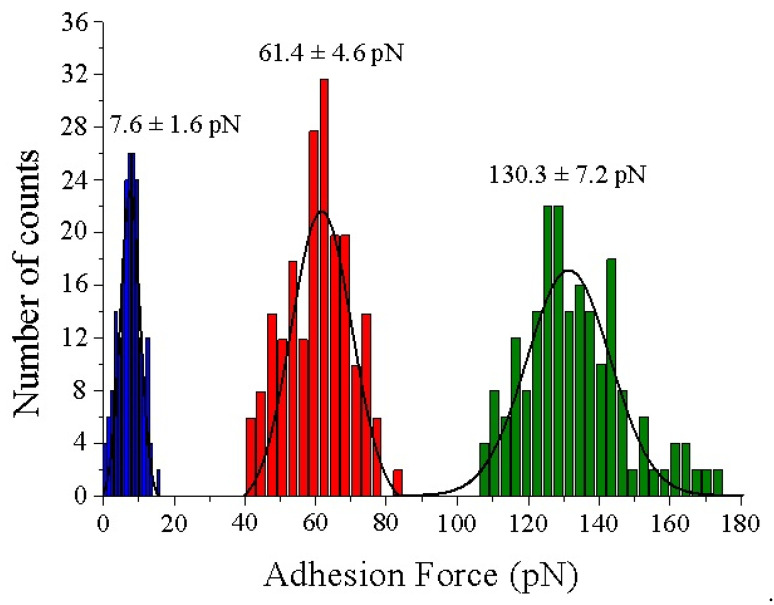
Force histograms analysis and Gaussian fitting for the specific adhesion forces found for the complexes formed between the thiol layer (blue), avidin (red) and streptavidin (green) with biotinylated tips, respectively. N 200 for each condition.

In the topography images of Figure 4 and Figure 5, the lateral resolution is not as good as when using bare tips because they are obtained with functionalized tips. This aspect adds some diffusion induced by the free movement of biotin bound to the flexible PEG molecules during scanning in liquid medium. To optimize the gathered interaction force data, the experiments have been conducted at scan rates of 130 pixels/s to prevent dragging force effects (F_drag_). The appearance of non-specific tip-sample forces at high acquisition velocities (upper than 250 pixel/s) has been demonstrated previously [38]. The insets of Figure 4 depict a good correlation between topography and adhesion maps due to the absence of detrimental unspecific tip-sample adhesion events. Statistical analysis of the adhesion force events formed between the biotinylated AFM tips and the functionalized mica surfaces reveal that the mean value of the specific interactions given by the rupture of streptavidin–biotin complexes is considerably higher, slightly more than two times, than that found for avidin–biotin complexes (130.3 ± 7.2 pN vs. 61.4 ± 4.6 pN, respectively) (Figure 6). The thiol layers render negligible interactions with biotin (7.6 ± 1.6 pN). The Gaussian distributions exhibit a broader range of adhesion forces for the streptavidin–biotin complex ruptures, comprised from 107.5 pN to 173.5 pN, while avidin–biotin rupture forces and biotin–thiolated mica adhesions range from 41.5 pN to 83.5 pN and 0.5 pN to 15.5 pN, respectively. These results are in line with previous studies [38,47,48,49].

The numerous chemical bonds involved in strept(avidin)–biotin biosystems and the dependence of unbinding forces regarding loading rates (R, applied force per time unit) may lead to different adhesion force results shown in other works [50]. The great complexity of strept(avidin)–biotin systems and the heterogeneity in the unbinding pathway may be explained by the existence of nearly different isoenergetic local minima [51] that are undergone in the energy landscape [52]. Other aspects that could impact on the strept(avidin)–biotin interaction are the conditions of image acquisition, such as ionic strength, pH [53], or the protein immobilization strategy used. In this last case, alternative protein chemical attachments may have an impact on protein motion during image acquisition. It has also been postulated that the stiffness of the molecular linkers that tether the protein to the solid surface could alter the unbinding complex process [54]. To shed light on this topic, different experimental and computational studies have been carried out [55].

Figure 5 clearly shows the discrimination in terms of the adhesion force of strept(avidin) hybrid mixtures. Black and red circles indicate the specific recognition events corresponding to avidin–biotin and streptavidin–biotin complex ruptures, respectively (Figure 5b). The specific adhesion force maps allow to quantify the ratio of protein entities present on the functionalized mica surface, being 46.5% and 42.0% for avidin and streptavidin proteins, respectively (N 200). This finding is not surprising taking into account that the mica surfaces were incubated with an equimolecular hybrid amount of avidin and streptavidin proteins. In addition, only 11.5% of protein features are of an unknown nature (Figure 5b, black circles).

## 4. Discussion

Atomic force microscopy has achieved great success in interrogating the physico-chemical properties of matter at a nanoscale level. In the frame of molecular recognition imaging, great efforts have been made to detect biomolecules of different chemistry present in a mixture. JM emerged as a suitable operational AFM mode to image biological samples in fluid. Implementing operating JM conditions under very small loaded forces in a repulsive regime allows the simultaneous mapping of specific rupture forces between two biomolecules, enabling a high degree of correlation of the topographic data with force spectroscopy information. The applied forces required are in the order of 20–50 pN. Here this value was optimized running several Fz curves (N 10) at different points of the sample; the selected force must allow operation under the repulsive electrical double layer (REDL) [37]. JM is an optimum force mode that allows good control in the set loaded forces. Here, optimization was achieved applicating 35 pN, scanning with the functionalized tips in repulsive regime being very stable for hours in liquid operation. Therefore, this is a stable method that not only provides qualitative recognition maps to locate specific receptors as other but also gives quantitative data on rupture forces of the involved complexes directly while avoiding undesired nonspecific adhesions that cloud all FS experiments. Here, an attempt has been made to take this method to the challenge of being able to distinguish between protein molecules exhibiting very similar properties and find the conditions to be able to locate and identify them through interaction with the same sensor, which has been successfully achieved with nanometer precision as a proof of concept. Both proteins have similar dimensions but display different adhesion properties scanned with the biotin ligand, and the exerted unbinding forces of the streptavidin–biotin complex are above two-fold times greater than the avidin–biotin complex at the selected working conditions, which need to be previously calibrated and set. The main drawback of the present methodology may be in those samples where the intermolecular rupture forces displayed between the functionalized AFM tip sensor and the different components of the biomolecular mixture are similar, and distinguishing the ones of the protein of interest is complicated. In these cases, the following careful assessments must be carried out: (i) find the operating conditions, such as the loading rate, that provide the unbinding force ranges that allow discrimination between ligand–biomolecule adhesion events produced during scanning; or (ii) use alternative ligand sensors with different chemistry that produce higher contrast in terms of adhesion events. We believe that this work provides a method that allows to locate biomolecules on substrates or on real micro-organism membranes, whose conditions require to be previously calibrated and implemented in order to be a reliable and effective method. It is hoped that this methodology can open a new gate in the generation of highly sensitive biosensors for a broad range of applications such as toxin detection, pathology monitoring, and electrochemical impedance genosensors [56], among others. Moreover, and based on the capability to form strong bonds with long-lasting interactions, for strept(avidin)–biotin complexes, a panoply of biotechnological applications may be designed based on adhesion properties, inspired by those developed in the last years in immunodetection [57], detection signal amplifiers [58], and nanomaterials purification [59].

## Figures and Tables

**Table 1:** Mean values of the ultraviolet–visible spectroscopy measurements of the negative controls assayed at a wavelength of 450 nm. Mica pieces of 1.0 cm^2^ for all the experiments were used. Error is below 5%.

	Control with Mica without Protein	Control of HRP–Biotin without TMB		
		Without Protein	Avidin 2 μg	Streptavidin 2 μg
Absorbance 450 nm	0.6367	0.0030	0.0024	0.0032

**Table 2:** Relationship between incubated micrograms of strept(avidin) with absorbances at 450 nm wavelength, and subsequently, immobilized strept(avidin) molecules per area unit of mica. Note that all strept(avidin) samples were diluted 10 times to not saturate the absorbance signal. Error is below 5%.

Incubated Protein (µg)	Avidin Absorbance (450 nm)	Avidin Molecules/cm^2^	Streptavidin Absorbance (450 nm)	Streptavidin Molecules/cm^2^
0.0	0	0	0	0
0.5	0.1458	1.26 · 10^10^	0.2051	7.91 · 10^10^
1.0	0.3267	2.83 · 10^10^	0.3308	1.27 · 10^11^
2.0	0.6422	5.56 · 10^10^	0.5938	2.28 · 10^11^
4.0	0.7635	6.61 · 10^10^	0.6838	2.63 · 10^11^
5.0	0.7943	6.87 · 10^10^	--------	--------
15.0	0.8495	7.35 · 10^10^	0.8895	3.42 · 10^11^

## Data Availability

Data contained within the article.
